# Enhancing genomic predictive ability of yield and yield-related traits in spring wheat by integrating major plant adaptation genes as a fixed effect

**DOI:** 10.1007/s00122-025-05075-8

**Published:** 2025-11-04

**Authors:** Yaotian Gao, Guriqbal Singh Dhillon, Pabitra Joshi, Justin Wheeler, Amandeep Kaur, Jianli Chen

**Affiliations:** Department of Plant Sciences, University of Idaho Aberdeen Research and Extension Center, Aberdeen, ID USA

## Abstract

Genomic selection (GS) has emerged as a promising approach in plant breeding, especially for complex traits like grain yield that are influenced by many small-effect quantitative trait loci (QTLs). Choosing the optimal prediction model is important for GS. Using 4 years of field data from a diverse panel of spring wheat lines, the present study assessed the ability of seven statistical models to predict grain yield (YLD), total spikelet number per spike (tSNS), thousand kernel weight (TKW), plant height (PHT), and heading date (HD). The Reproducing Kernel Hilbert Spaces (RKHS) model was used as a basis for comparing predictive ability improvement of the other six models when major plant adaptation genes controlling flowering time, photoperiod response, plant height, and vernalization were treated as fixed effects. Incorporating fixed effects into the model substantially improved genomic predictive abilities, increasing them by 13.6% for YLD, 19.8% for tSNS, 7.2% for TKW, 22.5% for HD, and 11.8% for PHT. Thus, wheat breeders could apply this approach to GS in cultivar development and improvement. This finding could also be applied for GS in other cereal crops. To our knowledge, this is the first demonstration in spring wheat of integrating adaptive marker sets (*FT*/*Ppd*/*Rht*/*Vrn*) as fixed effects within an RKHS framework to improve genomic prediction across yield and four yield-related traits.

**Supplementary Information:**

The online version contains supplementary material available at 10.1007/s00122-025-05075-8.

## Key message

Genomic predictive abilities in spring wheat for grain yield, total spikelet number per spike, thousand kernel weight, plant height, and heading date were significantly improved when major plant adaptation genes controlling flowering time, photoperiod response, plant height, and vernalization were integrated as the fixed effect in a selected base model RKHS.

## Introduction

Wheat (*Triticum aestivum* L.) is one of the most important cereal crops worldwide, providing around 20% of the global caloric intake (Shiferaw et al. 2013). However, both natural and anthropogenic factors are posing significant threats to global food security. An analysis of wheat breeding data in North America from 1960 to 2018 suggests that the current pace of variety improvement may be insufficient to overcome the negative impacts of climate change on wheat production (Zhang et al. 2022). Given these challenges, increasing wheat yield remains a critical wheat breeding objective.

Yield is a complex trait influenced by multiple genetic, environmental, and agronomic factors. Identifying yield-related traits and targeting them for improvement could enhance efforts to improve yield (Isham et al. 2021). Yield components, such as spikelet number per spike and thousand kernel weight, typically show higher heritability than grain yield itself (Wang et al. 2018; Zhang et al. 2018). Plant height is another key trait closely associated with plant architecture, lodging resistance, and yield performance (Wang et al. 2017). Additionally, heading date influences wheat adaptation to different environments, thus affecting yield (Chen et al. 2022). Therefore, a comprehensive understanding of yield and yield-related traits and their genetic basis would assist development of high-yielding wheat varieties through targeted breeding.

The phenotypic evaluation of yield-related traits is time-consuming and labor-intensive. Conventional breeding methods, including multi-location field testing and phenotypic selection, remain essential components of wheat breeding programs (Ahmar et al. 2020). However, this approach has several limitations. Field trials require land, labor, and time, and generally 10–20 years are needed to develop a new wheat variety (Haile et al. 2021; Cha et al. 2023). Also, phenotypic data collection requires large-scale manual measurements, often uses subjective assessments, and is influenced by environmental factors (Ahmar et al. 2020; Cha et al. 2023). These limitations highlight the need for more efficient and cost-effective approaches to accelerate the wheat breeding process and improve the accuracy of trait evaluation.

Advances in molecular breeding helped to overcome the limitations of conventional breeding methods, and marker-assisted selection (MAS) emerged as the first effective tool using molecular markers in plant breeding. MAS is a breeding approach that selects target genes based on molecular markers linked to desired traits, allowing for rapid and accurate identification of individuals possessing favorable alleles. According to Song et al. (2023), MAS offers several advantages over traditional phenotypic selection, including enhanced breeding efficiency, accelerated pyramiding of multiple allelic traits, and the ability to select for multiple quantitative trait loci (QTLs) or genes simultaneously. It also enables efficient selection of recessive genes without phenotypic evaluation in each generation and facilitates selection under conditions where phenotypic screening is difficult (Bradbury et al. 2005; Collard and Mackill 2008; Xu and Crouch 2008). However, despite these advantages, MAS has limitations when dealing with complex quantitative traits controlled by multiple genes with small effects. It often requires the development of biparental mapping populations, which is time-consuming and resource-intensive, and the effects of small-effect QTLs can be unstable across different environments and genetic backgrounds, necessitating multiple validations (Poland and Rutkoski 2016a; Crossa et al. 2017). As the complexity of target traits increases, it is necessary to adopt more advanced methods, such as genomic selection (GS), to overcome the limitations of MAS.

GS is a promising approach to overcome the limitations of MAS, especially for complex traits influenced by numerous small-effect quantitative trait loci (QTLs) (Bernardo 1994; Meuwissen et al. 2001). GS uses genome-wide markers to capture the genetic relationships among individuals, enabling the evaluation of complex traits controlled by multiple genes (Zhang et al. 2007). When traits are controlled by many QTLs each contributing a small proportion of phenotypic variation, GS is more effective than MAS (Plavšin et al. 2021). The GS process typically involves a training population and a breeding population. First, phenotypic and genotypic data from the training population are used to train prediction models. Subsequently, the trained model and genotypic data of the breeding population are used to calculate the genomic estimated breeding values (GEBVs) for the individuals in the breeding population (Meuwissen et al. 2001; Habier et al. 2007; Larkin et al. 2019). Finally, GEBVs for individuals within the breeding population can be used for selection, even in the absence of phenotypic information (Heffner et al. 2011; Xu et al. 2020). GS has been successfully applied in wheat breeding for various objectives, including yield improvement (Dreisigacker et al. 2021), disease resistance (Poland and Rutkoski 2016b), and quality traits (Joshi et al. 2024), and also has been explored for the domestication of new crops (Rasheed and Xia 2019). As pointed out by Heffner et al. (2009), GS has the potential to accelerate breeding cycles, increase genetic gain per unit time, and significantly improve the efficiency of breeding by reducing the reliance on extensive phenotyping.

Researchers have explored integrating MAS with GS for complex quantitative traits, particularly by incorporating markers or major-effect QTLs as fixed effects into GS models. This approach leverages the precise selection advantages of MAS for major genes with the comprehensive genomic information used in GS, thereby enhancing predictive ability. For example, Sarinelli et al (2019) found that including SNPs associated with heading date, plant height, and resistance to powdery mildew as fixed effects in GS models significantly increased predictive ability for these traits across different winter wheat training population sizes. Other research demonstrated that fitting large-effect markers as fixed effects enhances GS accuracy in various crops, such as pepper (Kim et al. 2022) and maize (Li et al. 2019). However, studies focusing on yield-related traits in wheat are relatively scarce. Further investigation is needed to assess the potential of this method in wheat breeding to improve predictive ability and breeding efficiency.

The present study in spring wheat assessed the genomic predictive ability of various parametric and semi-parametric models in predicting plant height (PHT), total spikelet number per spike (tSNS), heading date (HD), thousand kernel weight (TKW), and yield (YLD). We compared seven statistical models—ridge regression (RR), Bayesian ridge regression (BRR), reproducing kernel Hilbert space (RKHS), genomic best linear unbiased prediction (GBLUP), least absolute shrinkage and selection operator (LASSO), support vector machine (SVM), and random forest (RF)—to determine the optimal model for each trait (Hoerl and Kennard 1970; Cortes and Vapnik 1995; Tibshirani 1996; Breiman 2001; Meuwissen et al. 2001; Gianola et al. 2006; Habier et al. 2007; Long et al. 2011; de Los Campos et al. 2013a). The optimal model was used to test if incorporating major genes as fixed effects enhanced predictive ability. Unlike prior studies that focused on winter wheat, we test trait-specific combinations of adaptive genes (*FT*/*Ppd*/*Rht*/*Vrn*) as fixed effects in spring wheat and quantify gains over a 90K-SNP baseline RKHS model. This research aims to facilitate precise breeding for improved yield and yield-related traits in spring wheat.

## Materials and methods

### Plant materials

This study used 250 spring wheat varieties and elite lines (Supplemental Table 1). The collection was developed by breeding programs in the Northwestern Pacific region of the United States and the International Maize and Wheat Improvement Center (CIMMYT, Mexico City, Mexico). It encompasses three market categories of spring wheat cultivated in the Americas: soft white, hard white, and hard red. Most of the lines (> 50%) have been used in variety development programs for the region.

### Phenotypic evaluation

The panel was evaluated across four environments, designated as 2017–2023, with each year considered a distinct environment. The field trials were conducted at Aberdeen, Idaho located at 42°56′36" N and 112°50′22" W under irrigated conditions. Planting was typically conducted in early April, with harvesting occurring in late August. The field design was a randomized complete block with two replicates. Each genotype was planted in 3.0 m plots of seven rows with a row spacing of 21 cm. Yield goals were established for each wheat class based on historical yield data, and these goals were used to calculate optimal fertilizer applications. For hard spring wheat, the nitrogen requirement was calculated as 2.5 times the yield goal (bu/A), whereas for soft white spring wheat, it was 2.0 times the yield goal (Marshall et al. 2018). Management practices, soil fertility, and environmental conditions varied across the years, as summarized in Supplemental Table 2.

All traits were measured in trials 2022, and 2023. Yield was measured in 2017, while other traits were measured in 2021. Heading date (HD) was calculated from January 1st (Julian) until 50% of the plants had spikes emerging from the flag leaves. Plant height (PHT) was recorded as the height from the soil surface to the tip of the spike, excluding the awn at late maturity stage. Total spikelet number per spike (tSNS) was counted before harvest from ten randomly selected, fully developed spikes. Thousand kernel weight (TKW) was estimated by weighing 100 randomly selected seeds. Yield per acre (YLD) was evaluated by harvesting 4.5 square meters from each plot.

### Sparse testing methods for the allocation of lines to environments

**Fig. 1:**
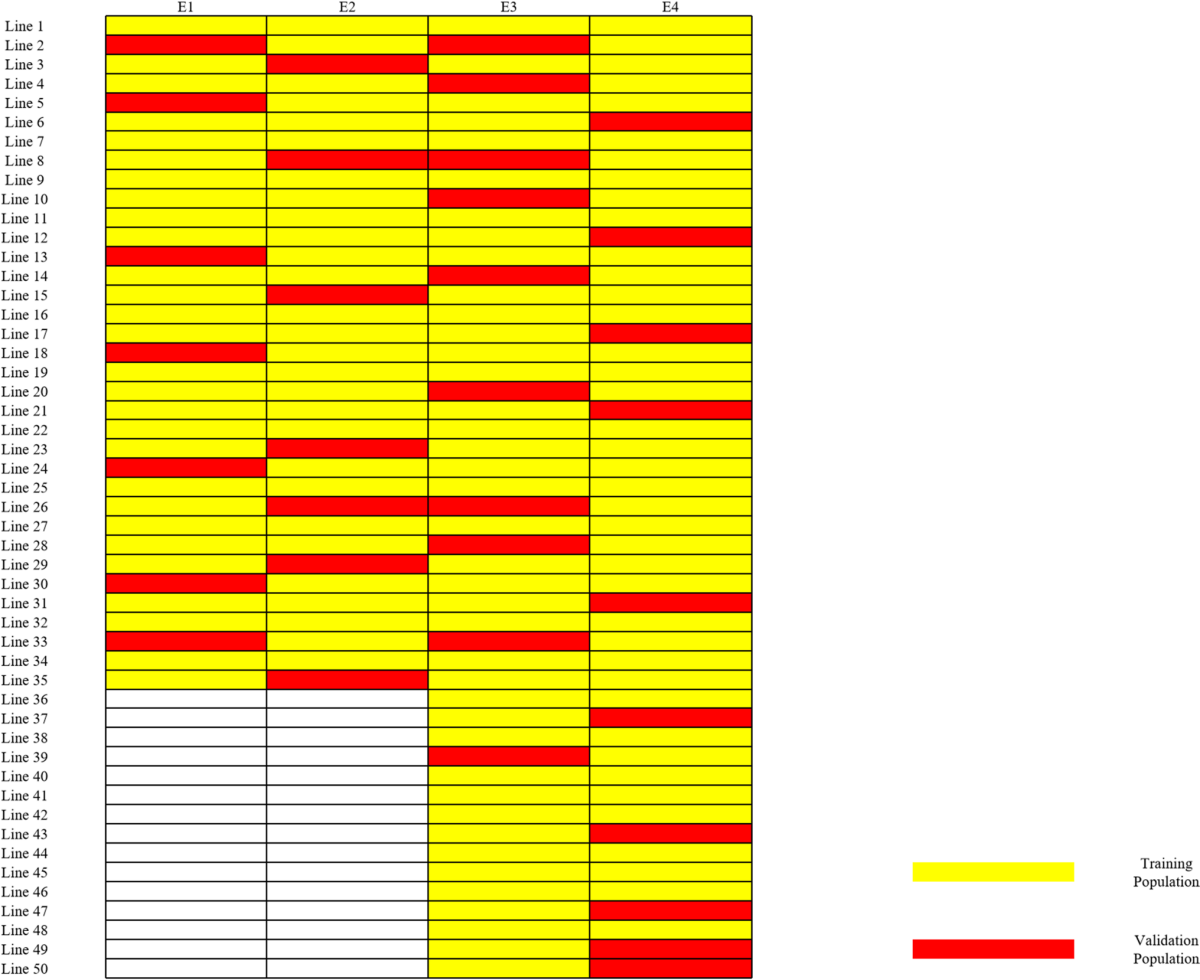
Sparse testing design with ten lines and four environments. The yellow squares represent the lines evaluated in each environment, while the red squares indicate the lines used as the Validation Population, and the white squares indicate the lines not included in the trials for those environments. In environments 2017 and 2021, a subset of 170 lines was selected from the full panel of 250 lines to represent the full range of breeding programs and market classes present in the panel, thus ensuring genetic diversity while reducing the experimental size. In environments 2022 and 2023, all 250 lines were evaluated. In each environment, 20% of the lines were used as the Validation Population, and 80% were used as the Training Population

A sparse testing approach was used to optimize resource allocation and statistical efficiency. In the initial trials (2017 and 2021), approximately 70% of the lines from the entire panel were strategically selected for evaluation (Fig. 1). This subset was chosen to represent the genetic diversity of the full panel while reducing the sample size. The data obtained from these initial trials provided information on the performance of the selected lines in those environments. This sparse testing strategy aimed to optimize resource allocation by focusing on a representative subset of lines in the early stages of the experiment while still maintaining the ability to evaluate the full panel in later trials. In the subsequent trials (2022, 2023), all lines were evaluated, allowing for a comprehensive assessment of the entire panel. By integrating the data from all environments using best linear unbiased predictors (BLUPs), we were able to leverage the genetic correlations among environments to predict the performance of lines that were not tested in the first two trials. From each trial, 20% of the individuals were randomly selected to form the validation population, with the remaining 80% constituting the training population using the five-fold cross validation method.

### Phenotypic data analysis

The analysis integrated replications through random effects linear models across different environments, employing META-R version 6.4 (Alvarado et al. 2020) and lme4 v1.1–37 (Bates et al. 2015). The model was:

$$Y_{ijk} = \mu + E_{j} + R_{i} \left( {E_{j} } \right) + G_{k} + G_{k} \times E_{j} + \varepsilon_{ijk}$$

${\text{where }}Y_{ijk}$ represents the trait of interest, μ is the overall mean effect, $E_{j}$ denotes the effect of the jth environment, $R_{i} \left( {E_{j} } \right)$ is the replicate effect of the ith replicate within the jth environment, $G_{k}$ signifies the effect of the kth genotype, $E_{j} \times G_{k}$ indicates the interaction between the jth environment and the kth genotype, and $\varepsilon_{ijk}$ is the error term for the ith replication, jth environment, and kth genotype. For calculating BLUPs to represent the stable genetic value of each line, the effects for $E_{j} , G_{k}$, and $G_{k} \times E_{j}$ were all treated as random. All effects except for the μ were considered random. It is assumed that the random effects are independent and normally distributed with a mean of zero and their respective variances: $G_{k} \sim N\left( {0,\sigma_{g}^{2} } \right),E_{j} \sim N\left( {0,\sigma_{e}^{2} } \right),\left( {GE} \right)_{jk} \sim N\left( {0,\sigma_{ge}^{2} } \right), R_{i} \left( {E_{j} } \right)\sim N\left( {0,\sigma_{r}^{2} } \right),$ and $\in_{ijk} \sim N\left( {0,\sigma_{ \in }^{2} } \right)$. For each genotype *k*, we extracted the genotypic main-effect BLUP across environments ($\hat{\mu }_{k}$). These $\hat{\mu }_{k}$ values were used as the response for training and evaluating the genomic prediction models.

The broad-sense heritability was computed using the formula:

$$H^{2} = \frac{{{\upsigma }_{g}^{2} }}{{{\upsigma }_{g}^{2} + \frac{{{\upsigma }_{ge}^{2} }}{{n_{Envs} }} + \frac{{{\upsigma }_{e}^{2} }}{{n_{reps} \times n_{Envs} }}}}$$

${\text{Here}},\; \sigma_{g}^{2}$ is the genotypic variance, ${\upsigma }_{ge}^{2}$ represents the genotype by environment variance, ${\upsigma }_{e}^{2}$ is the error variance, $n_{Envs}$ is the total number of environments, and $n_{reps}$ is the number of replications.

Additionally, across environments the least significant difference (LSD) and coefficient of variation (CV) were calculated from the across-environment model to facilitate the comparison of the genotypic BLUPs. The trait distribution across environments was assessed by visualizing BLUPs and trait values using ggplot2 v3.4.4 (Wickham 2016) in R v4.2.2 (R Core Team 2022).

### Correlation and principal component analyses

Correlation coefficients among traits across different environments, including BLUPs, were calculated using corrplot v0.92 (Wei and Simko 2021) in R v4.2.2 (R Core Team 2022). Bivariate correlation analysis was conducted to assess the impact of different environments on traits. Principal component analysis was performed using the "prcomp" function in R to identify the number of principal components required to explain the variation across different environments, including BLUPs.

### Genotyping and structure

The population was genotyped using Illumina's 90K iSelect SNP chip. Raw data was provided by the USDA/ARS Cereal Crops Research Unit in Madison WI and was analyzed using Genome Studio v2.0.5 (Illumina 2010). Polymorphic markers were identified as those with distinct clusters in Genome Studio and with a minimum distance of 0.20 between the polar coordinates of normalized theta intensities. Markers were further filtered based on missing data (> 10%) and minor allele frequency (< 5%) using TASSEL v5.2.89 (Bradbury et al. 2007). The chromosome density plot was generated using SRplot (Tang et al. 2023).

The population structure was investigated using principal component analysis (PCA) based on the genotypic data. PCA was performed using TASSEL v5.2.89 (Bradbury et al. 2007). The population was divided into seven groups according to source: University of Idaho (UI), Washington State University (WSU), University of California, Davis (UCD), CIMMYT, Montana State University (MSU), United States Department of Agriculture (USDA), and Other (commercial varieties and international germplasm). The first three principal components for all traits were examined to establish relationships among lines within the population. Analysis of variance (ANOVA) was conducted using the 'aov' function in v4.2.2 (R Core Team 2022) to determine the effect of population structure on phenotypic traits. The seven groups, representing the population structure, were treated as the explanatory variable. The proportion of phenotypic variance (R^2^) explained by the population structure was calculated for each trait.

### Genomic prediction model selection

To identify the optimal genomic prediction model for trait evaluation, seven statistical models were assessed: BRR, RR, RKHS, GBLUP, LASSO, SVM, and RF. The traits (HD, PHT, TKW, tSNS, YLD) were first evaluated using across-year genotypic BLUPs estimated from a multi-environment mixed model to ascertain the genomic predictive ability of each model. Predictive ability was quantified as the Pearson correlation between the observed BLUPs and the cross-validated predictions on held-out genotypes.

The predictive ability of the models was evaluated using a repeated five-fold cross-validation (CV) scheme. This scheme was specifically designed to assess the model's ability to predict the performance of entirely new genotypes, a scenario that mirrors practical breeding applications. To achieve this, the 249 genotypes were partitioned at the genotype level into five random folds. For each iteration, four folds (≈80% of genotypes) served as the training set, and the remaining fold (≈20% of genotypes) was used as the validation set. This partitioning strategy ensured that all phenotypic records for a given genotype, across all environments, were either entirely in the training set or entirely in the validation set, thereby preventing data leakage between them. To obtain robust and stable estimates, the entire five-fold CV procedure was repeated 50 times, each with a new random partitioning. The partitioning was performed randomly without any stratification by population structure or other factors.

The parameters and descriptions of the individual methods are as follows:

1. GBLUP

The GBLUP model is a foundational and widely used method in genomic prediction, selected here as a benchmark for its computational efficiency and effectiveness in modeling polygenic traits (Daetwyler et al. 2010). It operates under the assumption that traits are influenced by numerous genes with small, additive effects (VanRaden 2008; Goddard and Hayes 2009). The model was implemented using the kin.blup function from the rrBLUP package (Endelman 2011). This approach leverages a genomic relationship matrix (G), computed with the A.mat function. Prediction error variance (PEV) was calculated to estimate the reliability of predictions. The statistical model for GBLUP can be expressed as:

$$y = \mu + Zg + e$$

where y is the vector of phenotypes, μ is the overall mean, *Z* is an incidence matrix linking genotypes to phenotypes, *g* is a vector of genomic breeding values, and *e* is the residual error. The model assumes that the genomic values *g* follows a normal distribution $g \sim N\left( {0,G\sigma_{g}^{2} } \right)$, $e \sim N\left( {0,\sigma_{e}^{2} I} \right)$**,** where the covariance structure is defined by the genomic relationship matrix (G) and $\sigma_{g}^{2}$ is the additive genetic variance.

2. RR and LASSO

RR and LASSO were chosen as two classical penalized regression methods designed to handle high-dimensional genomic data (*p* >  > n) and mitigate overfitting (Hoerl and Kennard 1970; Tibshirani 1996). Both models estimate marker effects based on the general linear model equation:

$$y = \mu + X\beta + e$$

where y is the vector of phenotypes, μ is the overall mean, X is the marker genotype matrix, β is the vector of marker effects to be estimated, and e is the residual error.

The key difference lies in the penalty term they add to the residual sum of squares (RSS) during minimization. RR (Ridge Regression) minimizes the function $RSS + \lambda \mathop \sum \limits_{j = 1}^{p} \beta_{j}^{2}$, employing an L2 penalty. This approach assumes that most markers have small, non-zero effects, which are normally distributed, making it particularly suitable for complex polygenic traits.

In contrast, LASSO minimizes $RSS + \lambda \mathop \sum \limits_{j = 1}^{p} \left| {\beta_{j} } \right|$, using an L1 penalty. This is equivalent to assuming a double-exponential prior on marker effects and performs automated feature selection by shrinking the coefficients of less informative markers to exactly zero, making it ideal for traits potentially controlled by a smaller number of loci with larger effects.

RR and LASSO were implemented using the glmnet package (Friedman et al. 2010). For each main training fold, an inner cross-validation was performed to determine the optimal shrinkage parameter ($\lambda$). The $\lambda$ value that minimized the mean squared error within the training set was selected for prediction on the validation set.

3. RKHS and BRR

RKHS and BRR were implemented using the BGLR package (Pérez and de Los Campos 2014).

RKHS was chosen for its capacity to capture complex non-additive genetic effects, such as epistasis, without explicitly modeling them. It is a non-parametric method that transforms the marker data into a higher-dimensional space using a kernel function, allowing it to model non-linear relationships (Gianola et al. 2006). The model is defined as

$$y = \mu + u + e$$

where u denotes random genetic values with $u \sim N\left( {0,K\sigma_{u}^{2} } \right)$, and $e \sim N\left( {0,I\sigma_{e}^{2} } \right)$. The kernel matrix K was built with a Gaussian (radial basis) kernel, $K_{ij} = exp\left( { - hd_{ij} } \right)$, where $d_{ij}$ is the scaled squared Euclidean distance between the $i^{th}$ and $j^{th}$ genotype computed on the centered, unit-variance marker matrix; the bandwidth *h* governs smoothness and therefore the degree of nonlinearity captured by the model.

This model assumes that phenotypes arise from a smooth, possibly non-linear function of marker profiles captured by a positive-semidefinite kernel (here Gaussian), with Gaussian homoscedastic residuals and complexity governed by the kernel bandwidth (Jiang and Reif 2015). For RKHS, the bandwidth parameter (h) was set to 0.5.

BRR was included as the Bayesian analogue of RR, representing a standard method in genomic selection with strong empirical performance. Its primary advantages are its ability to provide full uncertainty quantification for all parameters and to perform adaptive shrinkage (de Los Campos et al. 2013a). The model is defined as

$$y = \mu + X\beta + e$$

where *y* is the vector of genotype responses, *X* is the centered and scaled marker matrix, and $e \sim N\left( {0,I\sigma_{e}^{2} } \right).$ Marker effects were assigned a common zero-mean normal prior, $\beta_{j} \sim N\left( {0,\user2{ }\sigma_{\beta }^{2} } \right)$, and the variance components ($\sigma_{\beta }^{2} ,\sigma_{e}^{2}$) received weakly informative priors and were estimated jointly with *β* via MCMC using BGLR. This model assumes a linear additive architecture in which all marker effects share a common zero-mean normal prior whose variance is estimated from the data, and residuals are independent and homoscedastic conditional on the variance components (de Los Campos et al. 2013a). For BRR, the model was run for 8,000 iterations with a burn-in period of 2,000 and a thinning interval of 3. Prior parameters were set as df_0_ = 5 and R^2^ = 0.5.

4. SVM

SVM (Support Vector Machine) was included as a powerful, non-parametric machine learning approach (Cortes and Vapnik 1995). It was chosen to explore its potential in capturing complex, non-linear patterns in the genotype–phenotype relationship (Zhao et al. 2020). The use of a kernel function, such as the Radial Basis Function (RBF), allows it to implicitly map the input data into a higher-dimensional feature space to model non-linearity (Bishop 2006). This approach assumes that, after standardization of inputs, the genotype–phenotype map can be well approximated by a smooth non-parametric function in the RBF-induced feature space, such that appropriate choices of kernel width (γ) and capacity (C, ν) capture the relevant non-linear structure while residual variation is centered at zero and exchangeable. The learned decision function takes the form

$$f\left( x \right) = \mathop \sum \limits_{i} (\alpha_{i} - \alpha_{i}^{*} )K\left( {x_{i} ,x} \right) + b$$

where $K\left( {x_{i} ,x} \right) = exp\left( { - \gamma \parallel x_{i} - x\parallel^{2} } \right)$, model capacity and sparsity are governed by the hyperparameters *C* and *ν.* SVM regression was performed using the e1071 package (Dimitriadou et al. 2008). Nu-regression was employed with a radial basis function (RBF) kernel. The cost parameter was set to 10, and the gamma parameter was set to 0.001 (Charmet et al. 2020).

5. RF

RF (Random Forest) was selected as another robust, non-parametric machine learning method (Breiman 2001). The rationale for its inclusion is its high predictive accuracy and its intrinsic ability to model complex interactions among predictors (i.e., epistasis) without requiring explicit definition (Heslot et al. 2012). It makes no assumptions about the distribution of the data and is generally robust to overfitting by aggregating the predictions from all individual trees (Breiman 2001).

RF was implemented using the randomForest package with default parameters (Liaw and Wiener 2002). These include growing 500 trees and setting mtry = *p*/3 for regression, where *p* is the number of predictors.

### Major genes KASP markers

All collected KASP markers were initially screened across 250 spring wheat varieties to identify polymorphic markers for subsequent analyses. Single nucleotide polymorphism (SNP) genotyping was performed using KBioscience's Competitive Allele-Specific PCR (KASP) technology. Genotyping assays were conducted using a CFX384 TouchTM Real-Time PCR Detection System (Bio-Rad, Hercules, CA) following the standard LGC Genomics protocol for reaction composition and PCR conditions. The final plate reading was performed at 25 °C, and genotype calling was accomplished using the allelic discrimination function in CFX Maestro software (Bio-Rad, Hercules, CA).

A set of KASP markers targeting major genes involved in key developmental and adaptive processes in wheat was used, including *FT* (*FTA2, FTB1, FTD1*), *Ppd* (*PpdA1a, PpdD1*), *Rht* (*Rht25, RhtB1, RhtD1*), and *Vrn* (*VrnA1, VrnB1*) gene families. The table (Supplemental Table 3) provides detailed information on each marker, including the specific alleles assayed and their known functional effects on phenotype.. These loci were selected based on their roles in controlling HD, tSNS, PHT, and ultimately YLD through both direct and indirect pathways. For example, *Rht* genes directly regulate stem elongation and thus plant height (Pearce et al. 2011; Mo et al. 2018) and *FT*, *Ppd*, and *Vrn* genes collectively shape flowering dynamics by influencing photoperiod and vernalization responses, which in turn affect HD and tSNS (Boden et al. 2015; Dragovich et al. 2021; Brassac et al. 2021; Chen et al. 2022). While *FT*, *Ppd*, and *Vrn* primarily exert indirect effects on yield by determining the timing of key growth stages and adaptation to environmental cues (Kamran et al. 2014), *Rht* can alter canopy structure and resource allocation, thereby influencing yield potential (Hayat et al. 2019; Jobson et al. 2019; Xu et al. 2023).

### Analysis of integrated marker effects

For the comprehensive genetic analysis, we employed a two-step approach to evaluate marker effects. Initially, the selected KASP markers were incorporated as random effects into the existing 90K SNP dataset. Subsequently, these markers were reclassified as fixed effects and integrated into the statistical model, while simultaneously removing their corresponding SNP data from the 90K dataset. The statistical modeling was implemented using BGLR (Pérez and de Los Campos 2014). The linear predictor can be represented by the following equation:

$$y = X\beta + Zu + e$$

Here *y* is the $n \times 1$ vector of responses; $X = \left[ {x_{0} ,x_{1} , \ldots ,x_{p} } \right]$ is the $n \times \left( {p + 1} \right)$ design matrix for fixed effects, with $x_{0} = 1$ the intercept column; $\beta = \left( {\beta_{0} ,\beta_{1} , \ldots ,\beta_{p} } \right)^{T}$ is the fixed-effect coefficients (including the overall mean and major gene markers, e.g., KASP); *Z* is the $n \times m$ design matrix for random genomic effects; $u = \left( {u_{1} , \ldots ,u_{m} } \right)^{T}$ are the random effects (genomic breeding values captured by the 90K SNP markers); and *e* is the $n \times 1$ vector of residuals. $u \sim N\left( {0,K\sigma_{g}^{2} } \right)$, $e \sim N\left( {0,I\sigma_{e}^{2} } \right)$.

### Comparison of different strategies for integrating major genes as fixed effects

To evaluate the effectiveness of incorporating major genes as fixed effects in GS models, we designed four distinct genotype combination strategies. Using the 90K marker set as the baseline model, we assessed three integration approaches: direct incorporation of 16 KASP markers into the marker matrix (90K + Major genes), inclusion of all 16 KASP markers each as individual fixed effects (90K + Major gene(fixed effect)), and utilization of trait-specific KASP marker combinations that demonstrated improved predictive ability in our previous analysis (90K + Selected Major gene(fixed effect)). The final strategy, '90K + Selected Major gene (fixed effect)', was developed by creating a trait-specific set of markers. For each of the five traits, we first evaluated each of the 16 KASP markers individually as a fixed effect. We then selected the subset of markers that showed a positive impact on predictive ability for that specific trait to form the combined fixed effect model. Additionally, we evaluated the impact of population structure as an additional fixed effect, using the first five principal components derived from both MDS and PCA dimensionality reduction methods.

### Climate data acquisition and processing

Daily meteorological data for the Aberdeen, Idaho weather station (USC00100031) was obtained from the National Centers for Environmental Information (NCEI) for the period of January 1, 2015, to December 31, 2024. The dataset included daily maximum temperature (T_MAX_), minimum temperature (T_MIN_), and precipitation (PRCP). Mean temperature was computed as

$$T_{mean} = \frac{{\left( {T_{MAX} + T_{MIN} } \right)}}{2}$$

To evaluate the climate conditions during the spring wheat growing season (approximately April–August), monthly climate summaries were generated. For each year from 2015 to 2024, the total monthly precipitation (mm) and the average monthly air temperature (°C) were calculated. A 10-year baseline (2015–2024) average was then computed for each month for both variables. The climate patterns of key experimental years (2017, 2021, 2022, and 2023) were visualized for the period by plotting their monthly values against the 10-year monthly average.

## Results

### Phenotypic evaluation

**Fig. 2:**
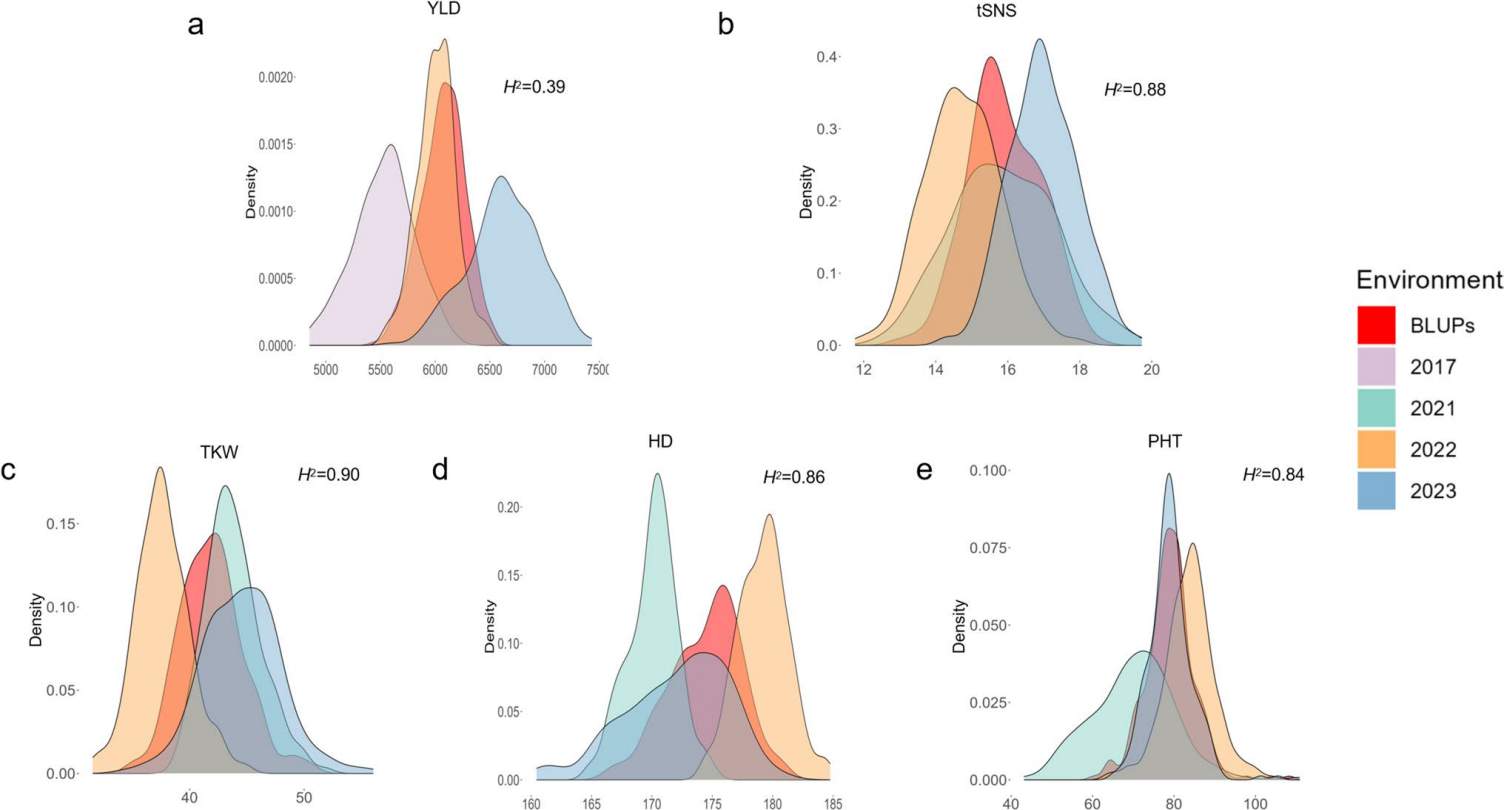
Distribution and broad-sense heritability (*H*^2^) of five agronomic traits in wheat across multiple environments. **b** total spikelet number per spike (tSNS), **c** thousand kernel weight (TKW), **d** heading date (HD) and **e** plant height (PHT) was evaluated in three environments (2021–2023), while **a** yield (YLD) was evaluated in three environments (2017, 2022, 2023). BLUPs represent the best linear unbiased predictions across environments

YLD was evaluated for its phenotypic variation across three environments. The results from the multi-environment randomized complete block trials showed that the BLUPs for YLD ranged from 5464 to 6534 kg/ha. The lowest YLD was observed in 2017 with a mean YLD of 5529 kg/ha, and highest in 2023 with a mean of 6631 kg/ha (Fig. 2a). YLD in 2023 was on average 1102 kg/ha higher than in 2017 (Supplemental Table 4).

The BLUPs for tSNS ranged from 12.89 to 18.57. The tSNS was lowest in 2022 with a mean of 14.75, and highest in 2023 with a mean of 16.99. An average of 2.24 more spikelets per spike occurred in 2023 compared to 2022 (Supplemental Table 4). The tSNS distribution showed relatively low phenotypic variability with a near-normal distribution. The variability of tSNS across environments was low and consistent (Fig. 2b).

The BLUPs for TKW ranged from 34.59 to 50.78 g. TKW was lowest in 2022 with a mean of 37.64 g, and highest in 2023 with a mean of 44.56 g. TKW in 2023 was 6.92 g greater than in 2022 (Supplemental Table 4). TKW data showed a right-skewed, leptokurtic distribution. The variability of data across environments was relatively low and consistent (Fig. 2c).

The BLUPs for HD ranged from 166.45 to 181.00. HD was earliest in 2021 with a mean of 170.08, and latest in 2022 with a mean of 179.21. HD in 2022 was 9.13 days later than in 2021 (Supplemental Table 4). The distribution of data revealed relatively low phenotypic variability in HD, with a near-normal distribution. The variability of data within each environment was also relatively low and consistent (Fig. 2d).

The BLUPs for PHT ranged from 60.44 to 108.30 cm. PHT was shortest in 2021 with a mean PHT of 70.09 cm, and tallest in 2022 with a mean PHT of 83.89 cm. PHT in 2022 was 13.80 cm taller than in 2021 (Supplemental Table 4). The distribution of data showed greater variability in PHT with a right-skewed, leptokurtic distribution. The variability of PHT data across environments was relatively low and consistent (Fig. 2e).

Heritability analysis revealed the relative contributions of genetic factors to the phenotypic variation of each trait. The broad-sense heritability for tSNS, TKW, HD, and PHT was 0.88, 0.90, 0.86, and 0.84, respectively, indicating that the phenotypic variation in these traits was mainly determined by genetic factors. However, YLD had a relatively lower heritability of 0.39, indicating that it is greatly influenced by environmental factors (Fig. 2).

### Correlation and principal component analysis

**Fig. 3:**
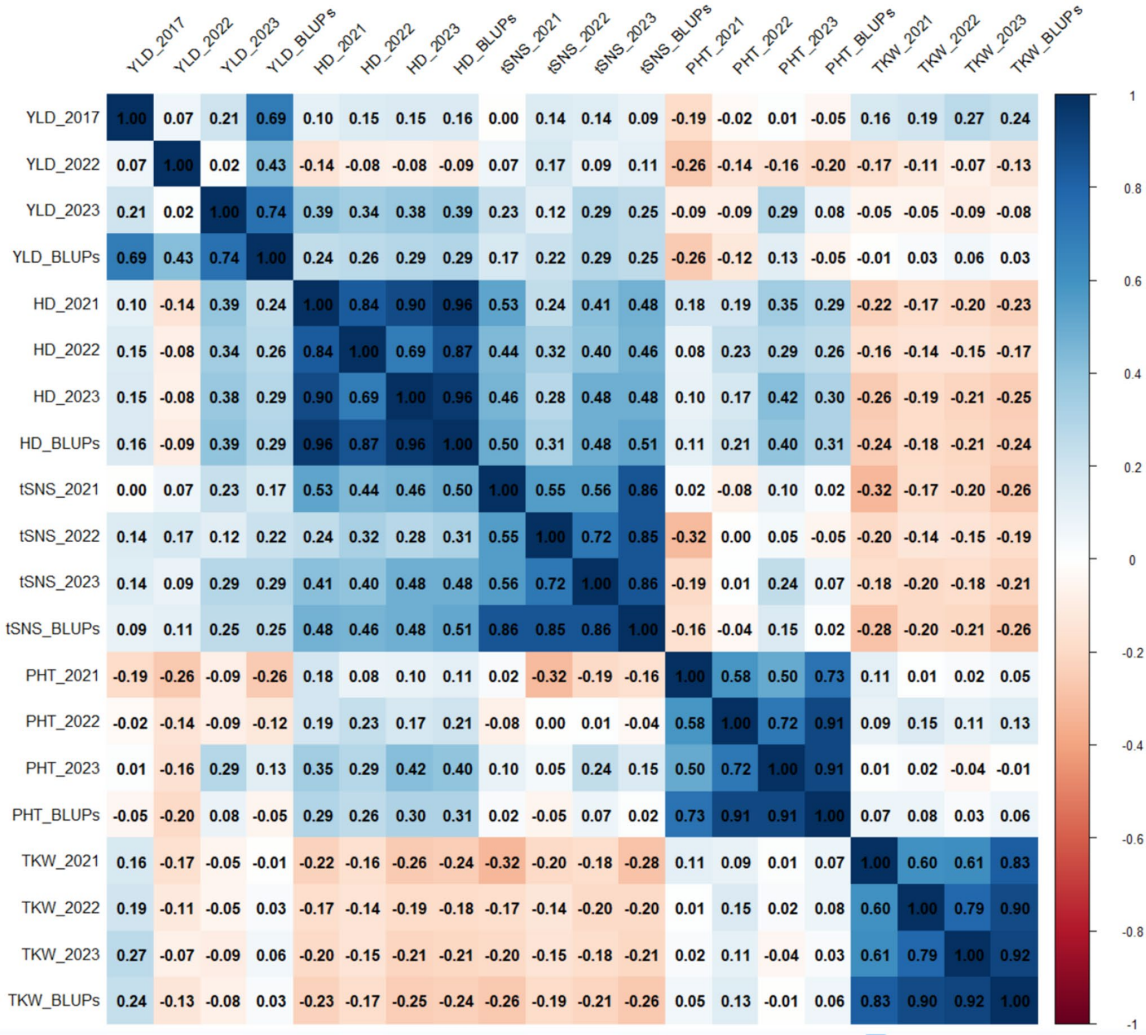
Correlation matrix of yield (YLD) and yield-related traits, including heading date (HD), plant height (PHT), thousand kernel weight (TKW), and total spikelet number per spike (tSNS), across multiple environments and their best linear unbiased predictions (BLUPs)

The correlation analysis revealed several relationships among the traits studied. The BLUPs values for each trait showed high correlations with the trait values across environments, indicating their suitability for integrating phenotypic data from multiple environments for subsequent genetic analysis. For instance, the BLUPs values for tSNS had correlation coefficients of 0.86, 0.85, and 0.86 with tSNS across 2021, 2022, and 2023, respectively. Similarly, the BLUPs values for TKW, HD, and PHT exhibited correlations above 0.7 with their corresponding trait values in most environments. The correlations between YLD BLUPs and YLD in some environments were slightly lower. YLD was positively correlated with tSNS (r = 0.25) and HD (r = 0.29). In contrast, YLD did not correlate with TKW (r = 0.03) and PHT (r = − 0.05), indicating that plant height and thousand-kernel weight are likely not primary yield-determining factors in this panel (Fig. 3).

**Fig. 4:**
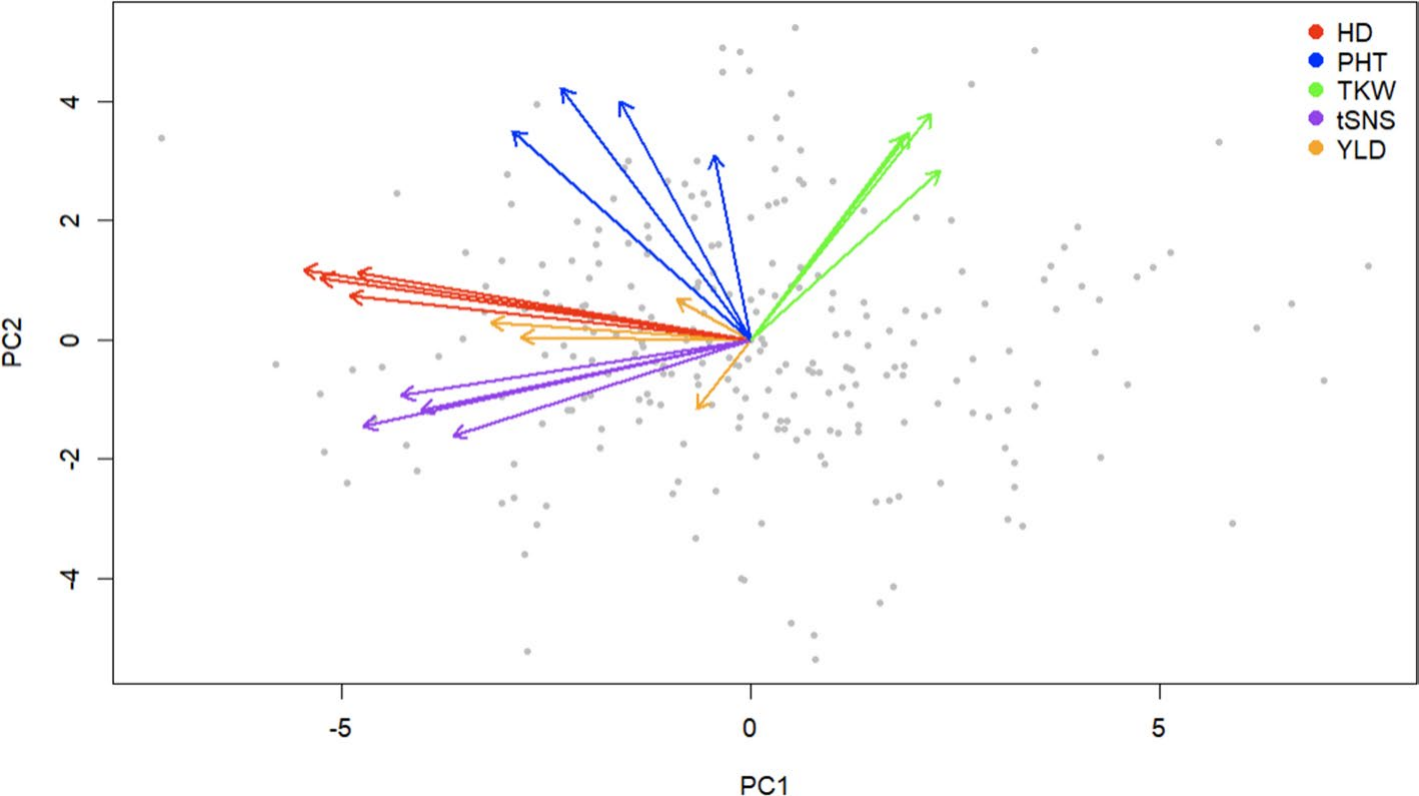
Principal component analysis (PCA) biplot of yield (YLD) and yield-related traits (HD, PHT, TKW, and tSNS) across multiple environments (2017–2023) and their BLUPs. Red arrows represent heading date (HD) in 2021–2023 and BLUPs, blue arrows represent plant height (PHT) in 2021–2023 and BLUPs, green arrows represent thousand kernel weight (TKW) in 2021–2023 and BLUPs, purple arrows represent total spikelet number per spike (tSNS) in 2021–2023 and BLUP, and orange arrows represent yield (YLD) in 2017, 2022, 2023, and BLUPs

The PCA biplot provided additional insights into the relationships between YLD and yield-related traits (tSNS, TKW, HD and PHT). YLD exhibited small angles with HD and tSNS, indicating possible positive relation among the traits and suggesting that HD and spikelet number per spike might be major factors influencing wheat yield in this panel (Fig. 4). In contrast, the near-90-degree angle between YLD and PHT indicated a weak relationship when the first two principal components were studied, suggesting that plant height is not a primary yield-determining factor. The obtuse angle between YLD and TKW indicated a negative relationship.

### Genotypic data and population structure

**Fig. 5:**
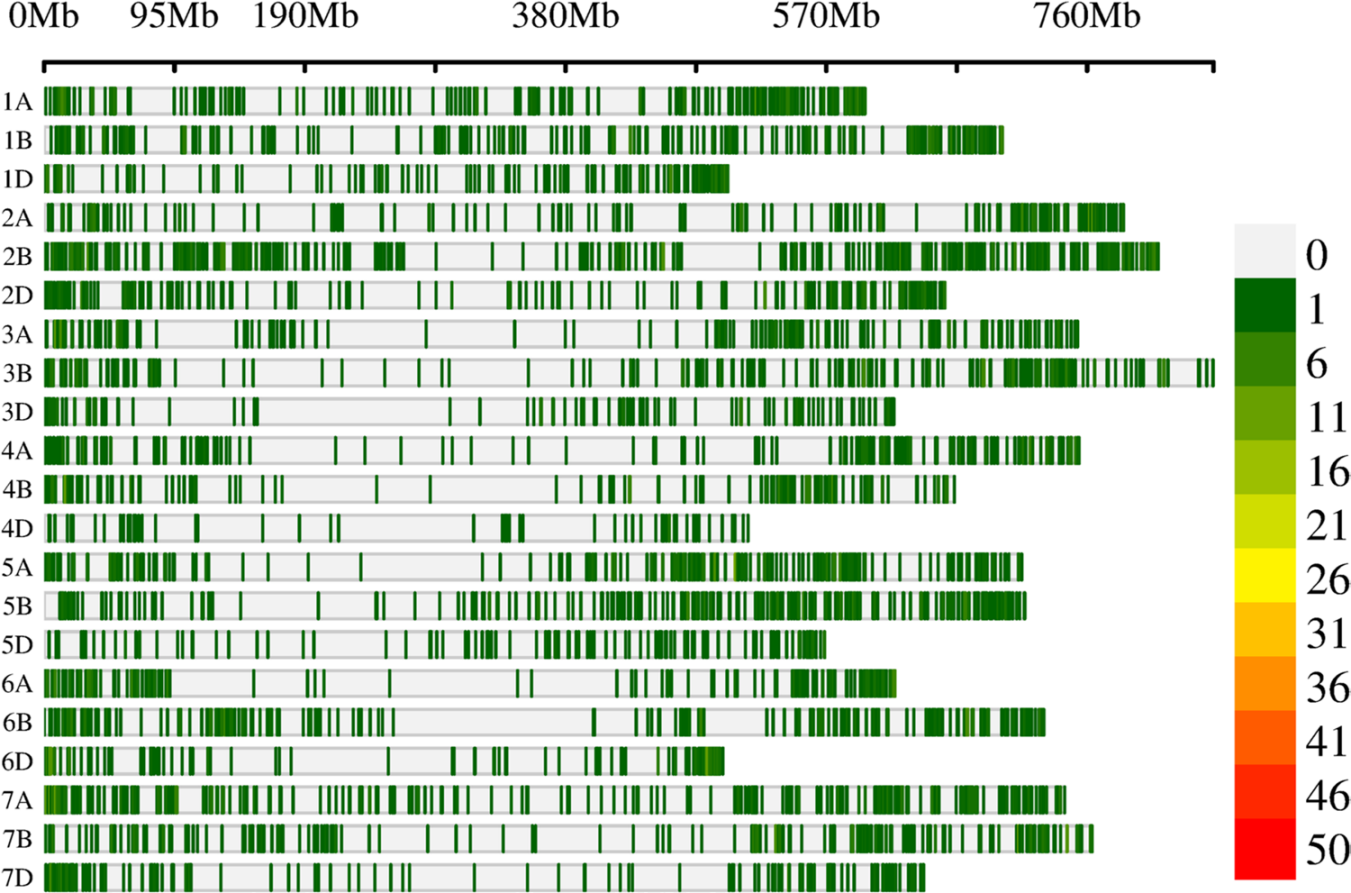
Wheat chromosomal SNP density distribution plot depicting the number of SNPs within 1 Mb window size. Chromosomes 1A–7A represent the A genome, 1B–7B the B genome, and 1D–7D the D genome. SNP positions and chromosome lengths are shown in megabases (Mb) based on the wheat reference genome sequence v2.1

After genotyping and filtering, 5,219 SNP markers (Supplemental Table 5) and 249 genotypes were retained and used for further analysis. The distribution of the SNPs on the reference genome of Chinese Spring RefSeqv2.1 showed 5,167 SNPs distributed across the 21 chromosomes and 52 located on unallocated scaffolds. The B genome had the most SNPs (2,201) followed by the A genome (1,903), while the D genome had the fewest SNPs (1,063) (Supplemental Table 6). Chromosome 2B had the most SNPs (508) and chromosome 4D had the least (62). SNP density and the average distance between adjacent SNP markers varied among different chromosomes (Fig. 5). Chromosome 2B had the highest SNP density (0.626 SNPs per Mb) and thus the smallest inter-SNP distance (1.597 Mb). Conversely, chromosome 4D had the lowest density (0.122 SNPs per Mb) and the largest inter-SNP distance (8.217 Mb). The B genome showed the highest SNP density (0.422 SNPs per Mb) and the smallest inter-SNP distance (2.368 Mb), while the D genome had the lowest density (0.267 SNPs per Mb) and the largest inter-SNP distance (3.745 Mb).

**Fig. 6:**
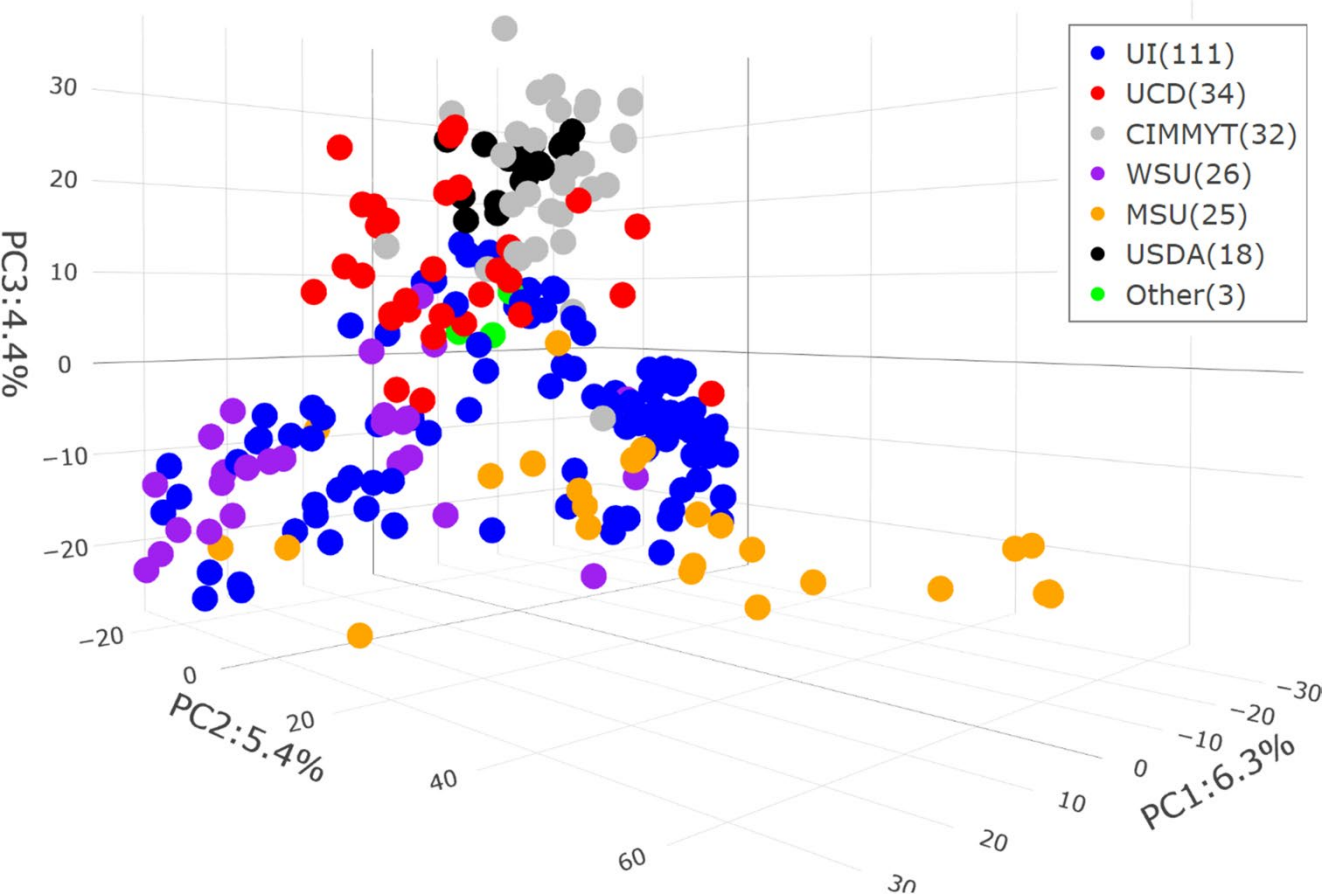
Three-dimensional PCA plot revealing the population structure of 249 spring wheat lines from multiple sources, including UI, UCD, WSU, CIMMYT, MSU, USDA, and other sources (commercial varieties and international germplasm.)

Principal component analysis (PCA) based on the genotypic data revealed a clear population structure among the 249 spring wheat lines (Fig. 6). The first three principal components (PCs) explained 6.3%, 5.4%, and 4.4% of the total genetic variance, respectively. The number of lines within each cluster ranged from 3 (Other) to 111 (UI). The largest cluster consisted of 111 lines from the UI, followed by 34 lines from UCD, and 32 lines from CIMMYT. ANOVA revealed that the population structure had significant effects on all five traits analyzed (*P* < 0.001) (Supplemental Table 7). However, the population structure accounted for only a modest portion of phenotypic variation; for example, it explained 11.8% of the yield variation. TKW showed the highest proportion of variance explained by the clusters (R^2^ = 24.1%), followed by PHT (R^2^ = 23.2%), YLD (R^2^ = 11.8%), tSNS (R^2^ = 10.7%), and HD (R^2^ = 7.6%). These results indicate that while population structure had a statistically significant effect, it explained a relatively modest portion of the overall phenotypic variance, particularly for yield.

### Comparison of genomic prediction models

**Fig. 7:**
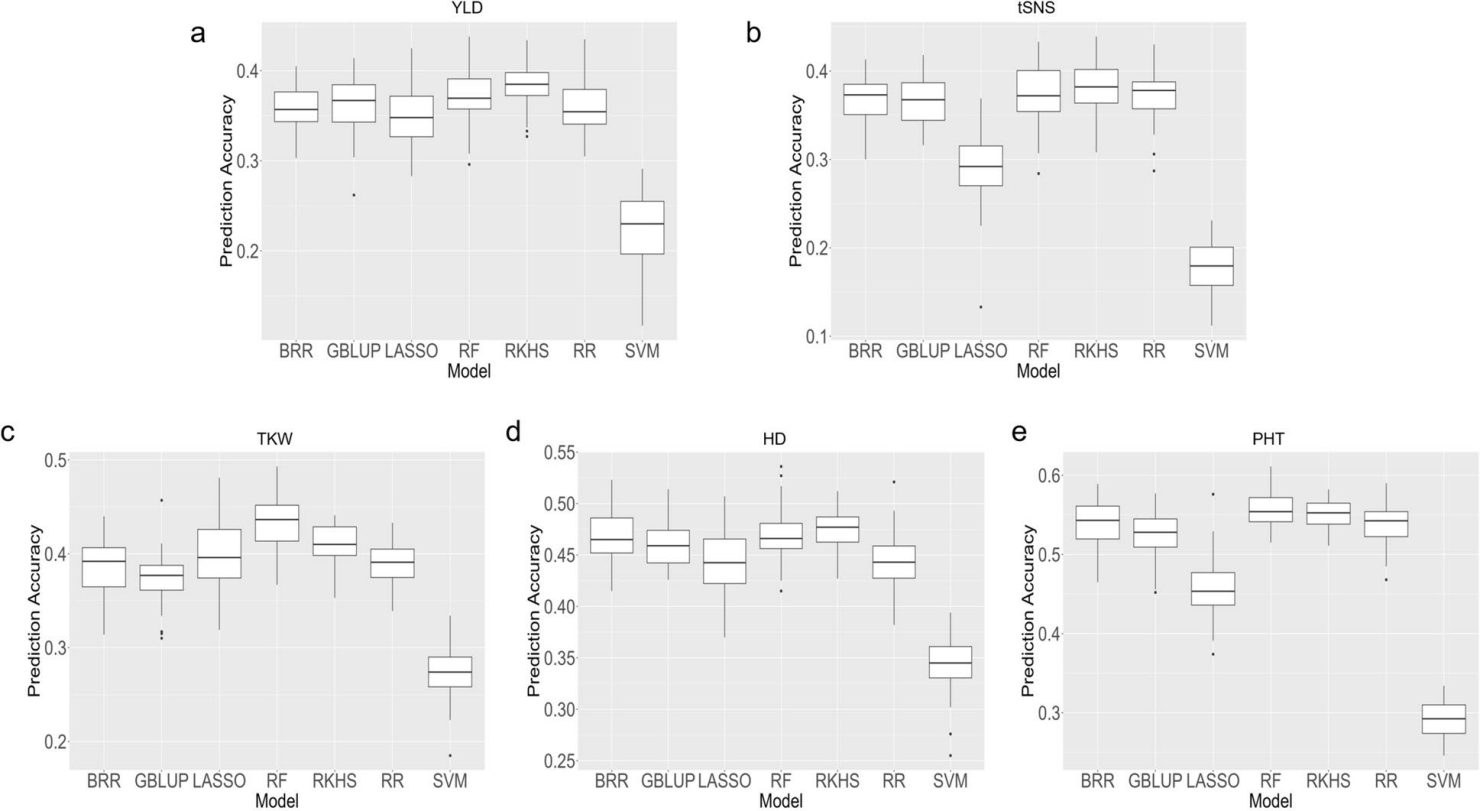
Boxplots depicting the predictive abilities of seven genomic prediction models (BRR, GBLUP, LASSO, RF, RKHS, RR, and SVM) for five agronomic traits in wheat based on the BLUPs of phenotypic data. The traits include **a** yield (YLD), **b** total spikelet number per spike (tSNS), **c** thousand kernel weight (TKW), **d** heading date (HD), and **e** plant height (PHT). The y-axis represents predictive ability, while the x-axis shows the different prediction models

The models BRR, GBLUP, RKHS, RF, RR, LASSO, and SVM, were evaluated using fivefold genotype-based cross-validation repeated 50 times, accuracies reflect prediction of untested genotypes to identify the model ability for predicting YLD, tSNS, TKW, HD, and PHT based on the BLUPs of phenotypic data. The SVM model consistently showed the lowest mean predictive abilities across all traits, ranging from 0.180 for tSNS to 0.345 for HD (Fig. 7, Supplemental Table 8). RKHS and RF demonstrated similar and superior performance compared to the other models, including the widely used GBLUP and RR, for all traits (Fig. 7). For YLD, tSNS and HD, RKHS outperformed RF.

Considering the comparable performance of RKHS and RF, we selected RKHS as the optimal model for genomic prediction because 1) RKHS demonstrated higher predictive ability and stability for more traits (YLD, tSNS and HD) compared to RF (Fig. 7) and 2) RKHS has shown high predictive abilities when applied in GS scenarios for yield prediction in wheat. For instance, the International Maize and Wheat Improvement Center (CIMMYT) successfully implemented RKHS for predicting grain yield in wheat, resulting in an estimated 7% genetic gain in yield when comparing the top 20% of lines with the bottom 20% of lines, as ranked by their GEBVs (Dreisigacker et al. 2021). These findings suggest that RKHS is a robust and effective approach for genomic prediction in wheat breeding programs.

Among the traits predicted using the RKHS model, PHT showed the highest median predictive ability (0.553), followed by HD (0.477), TKW (0.410), YLD (0.385), and tSNS (0.382) (Fig. 7, Supplemental Table 8). The difference in mean predictive ability between the most predictable trait (PHT) and the least predictable trait (tSNS) was 0.171, highlighting the varying degrees of predictability across the studied traits.

### Testing predictions across environments

All analyses here use single-environment, single-trait models; the across-year BLUP is treated as a pooled phenotype. Predictive ability is estimated under CV1 (new-line prediction) with repeated five-fold at the genotype level.

**Fig. 8:**
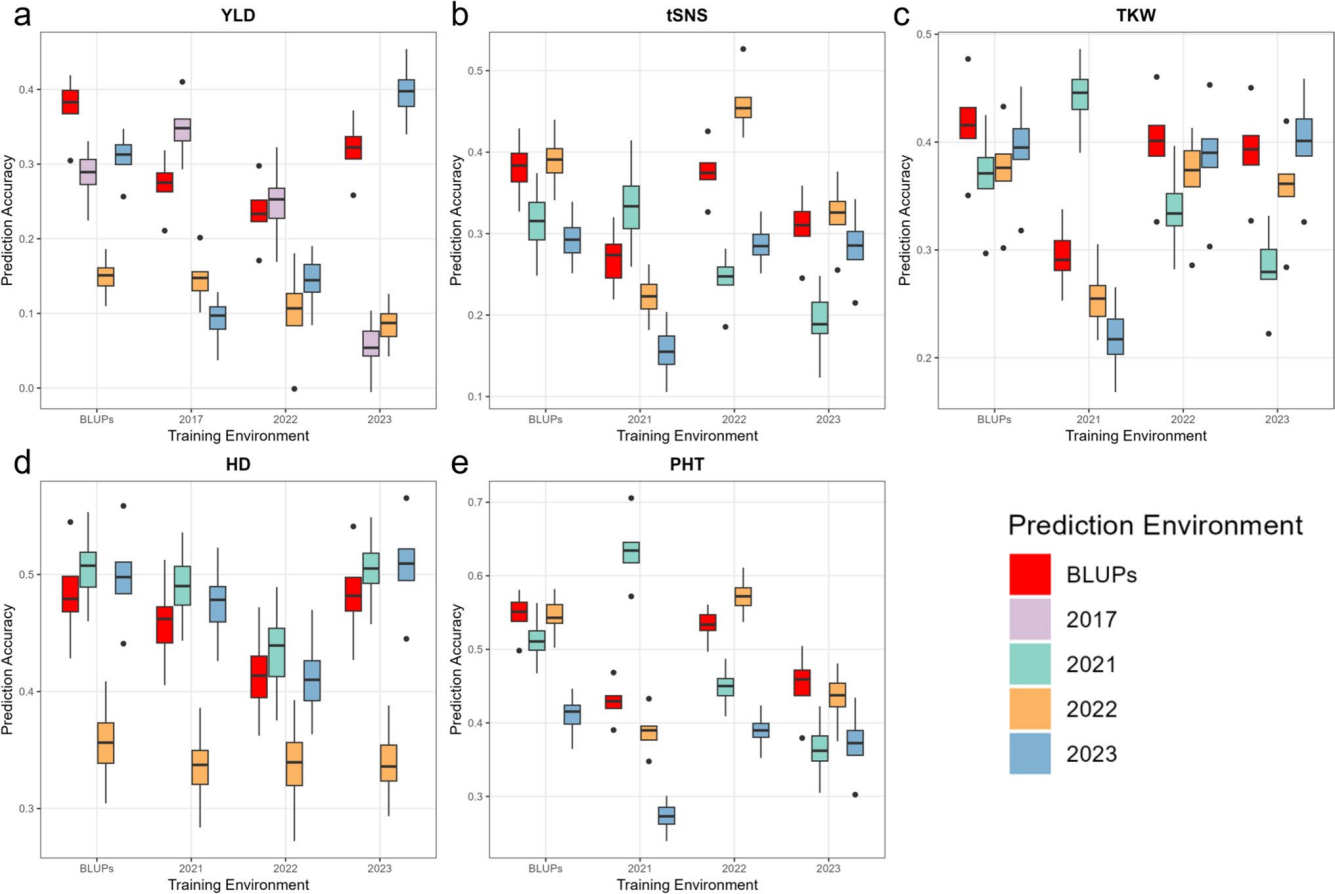
Comparison of predictive abilities for **a** yield (YLD), **b** total spikelet number per spike (tSNS), **c** thousand kernel weight (TKW), **d** heading date (HD), and **e** plant height (PHT), using models trained in different environments. The y-axis represents the predictive ability, and the x-axis shows the training environment for each model. The color of the bars indicates the predictive ability of the models in each testing environment. All results are based on the RKHS model

The predictive ability of yield-related traits and yield across different environments was studied using the optimal model (RKHS) and accuracies reported as median values. Single-environment models were generated by training and predicting within the same environment. For YLD, the predictive abilities were highest for a single-environment model was achieved when training and testing within 2023 (0.398). The model trained using the across-environment BLUPs also showed a high predictive ability of 0.383 when training and testing within the BLUP phenotype (Fig. 8a, Supplemental Table 9). For tSNS, the predictive abilities for a single-environment model were highest for environment 2022 (0.454) and BLUPs (0.384) (Fig. 8b). For TKW, the predictive abilities for a single-environment model were highest for environment 2021 (0.446) and BLUPs (0.416) (Fig. 8c). For HD, the predictive abilities for a single-environment model were highest for environment 2023 (0.509) and 2021 (0.490) (Fig. 8d). For PHT, the predictive abilities for a single-environment model were highest for environments 2021 (0.634) and 2022 (0.572) (Fig. 8e). BLUPs outperformed most of the evaluation environments.

As a sensitivity check of temporal extrapolation (single site across years), we also examined training–testing across environments; additional combinations are summarized in Supplemental Table 9. For different training–testing environment combinations, predictive ability ranges were 0.054–0.398 for YLD, 0.155–0.454 for tSNS, 0.217–0.446 for TKW, 0.336–0.509 for HD, and 0.273–0.634 for PHT (Fig. 8a–e). The respective ranges when using BLUPs to train the model were 0.151–0.383, 0.293–0.391, 0.371–0.416, 0.356–0.507, and 0.415–0.551 (Fig. 8). BLUPs were best suited for training models to predict values in other years at the same site (temporal extrapolation) (Supplemental Table 9).

Comparing the predictive abilities of yield and the yield-related traits across different training–testing environment combinations, the predictive abilities for the yield-related traits were generally higher than those for yield when using BLUPs to train the model.

### Integration of major genes as fixed effects in GS

**Fig. 9:**
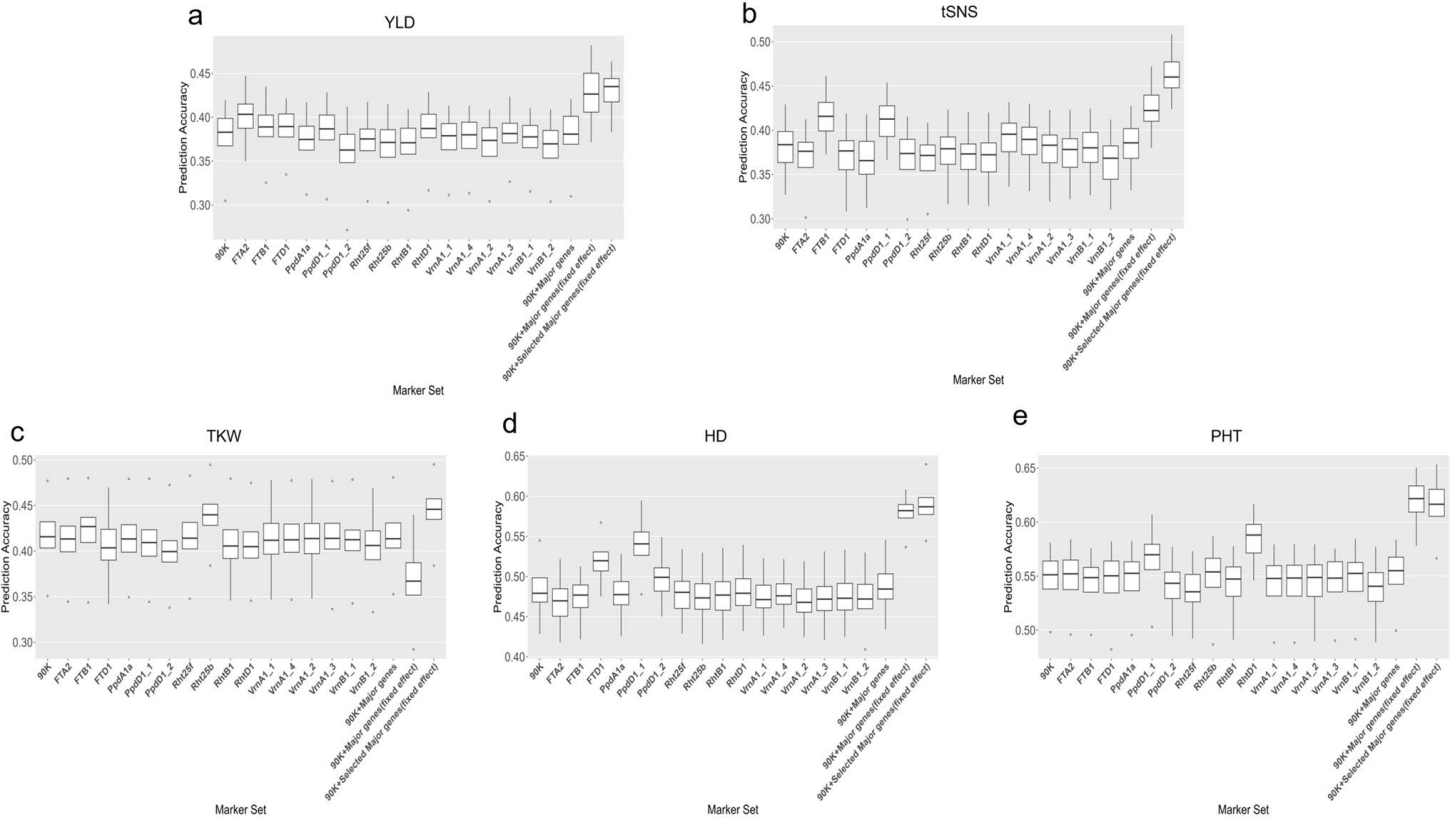
Predictive abilities of genomic selection models incorporating different marker sets as fixed effects for five traits. Predictive abilities were evaluated using RKHS models for **a** yield (YLD), **b** total spikelet number per spike (tSNS), **c** thousand kernel weight (TKW), **d** heading date (HD), and **e** plant height (PHT). The "90K" represents the baseline RKHS model without fixed effects. "90K + Major genes" includes direct incorporation of 16 KASP markers as random effects into the 90K SNP baseline. "90K + Major genes (fixed effect)" treats all 16 KASP markers as fixed effects in the model. "90K + Selected Major genes (fixed effect)" incorporates trait-specific KASP markers (bolded in the table) as fixed effects. Other markers indicate different genes used as fixed effects, including flowering time genes (*FT*), photoperiod response genes (*Ppd*), reduced height genes (*Rht*), and vernalization genes (*Vrn*). Predictive ability values represent the median predictive ability for each model-trait combination based on 50 repetitions

Using the RKHS model trained with BLUPs data as a basis for comparison, 16 markers for flowering time genes (*FT*), photoperiod response genes (*Ppd*), reduced height genes (*Rht*), and vernalization genes (*Vrn*), were tested as fixed effects (Fig. 9, Supplemental Table 3).

When used as fixed effects, some markers enhanced predictive ability for some traits (Supplemental Table 10). For YLD, the baseline predictive ability was 0.383, and several markers resulted in predictive abilities numerically higher than the baseline, including PpdD1_1 (0.387), RhtD1 (0.387), FTB1 (0.389), FTD1 (0.389), and FTA2 (0.403). For other traits, the inclusion of specific KASP markers resulted in predictive abilities that were numerically higher than their respective baseline models. For tSNS (baseline: 0.384), the highest abilities were achieved with FTB1 (0.416) and PpdD1_1 (0.413). Similarly, for TKW (baseline: 0.416), Rht25b (0.440) and FTB1 (0.427) yielded higher abilities. The predictive ability for HD (baseline: 0.479) showed a notable increase with PpdD1_1, reaching 0.541. For PHT (baseline: 0.551), RhtD1 demonstrated the greatest effect (0.588).

The different integration strategies showed varying effects on predictive ability (Fig. 9). Compared to the baseline model (90K), the direct incorporation of KASP markers (90K + Major genes) resulted in improvements for tSNS (0.386), HD (0.484), and PHT (0.555), but showed minor decreases in predictive ability for YLD (0.381) and TKW (0.414). When incorporating all 16 KASP markers as a collective fixed effect (90K + Major gene (fixed effect)), most traits exhibited substantial improvements in predictive ability, including YLD (0.426), tSNS (0.422), HD (0.582), and PHT (0.622). However, this strategy significantly decreased the predictive ability for TKW from 0.416 to 0.367 (Supplemental Table 10).

Among all evaluated strategies, the trait-specific KASP marker combination (90K + Selected Major gene (fixed effect)) had the best prediction performance. This approach improved predictive ability across most traits, achieving the highest values of 0.435, 0.460, 0.446, 0.587 for YLD, tSNS, TKW, and HD, respectively.

Comparing these results with the best single-gene fixed effects from our previous analysis, the 90K + Selected Major gene (fixed effect) strategy showed superior predictive power. This approach improved predictions as follows, YLD from 0.403 (FTA2) to 0.435, tSNS from 0.416 (FTB1) to 0.460, TKW from 0.440 (Rht25b) to 0.446, HD from 0.541 (PpdD1_1) to 0.587, and PHT from 0.588 (RhtD1) to 0.616. These results indicate that incorporating multiple trait-relevant major genes as fixed effects outperforms the single-gene fixed effect approach.

**Fig. 10:**
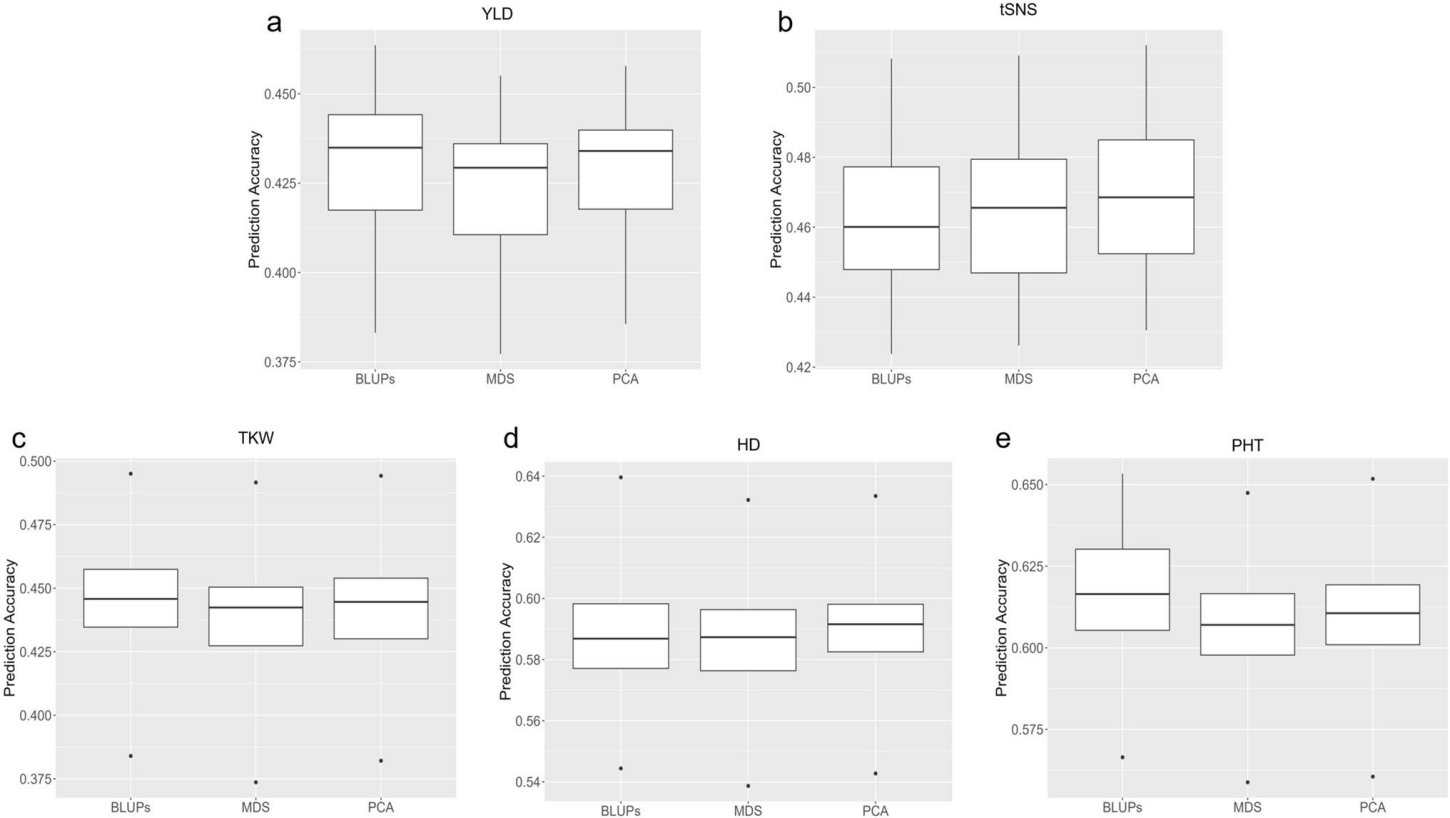
Predictive ability of genomic selection incorporating population structure for key agronomic traits in wheat **a** yield (YLD), **b** total spikelet number per spike (tSNS), **c** thousand kernel weight (TKW), **d** heading date (HD), and **e** plant height (PHT). "BLUPs" represents the strategy using "90K + Selected Major genes (fixed effect)" without incorporating population structure. "MDS" includes the first five MDS components to account for population structure. "PCA" incorporates the first five PCA components to account for population structure. Predictive ability values represent the median predictive ability for each model-trait combination based on 50 repetitions

Using the 90K + Selected Major gene (fixed effect) strategy, we further examined the influence of population structure as an additional fixed effect (Fig. 10). Both population structure approaches showed limited impact on predictive ability. Specifically, including a fixed effect for population structure in the prediction model changed accuracy by a maximum of 0.008 (ranging from − 0.009 to 0.008 across both MDS and PCA approaches). Although the PCA method demonstrated slightly better performance, the overall incorporation of population structure as an additional fixed effect did not significantly enhance predictive ability (Supplemental Table 11). These minor changes confirm that including population structure as an additional fixed effect did not meaningfully improve predictive ability in our models.

## Discussion

### Seasonal drivers of phenotypic variation in spring wheat

The significant phenotypic variation observed across the four experimental years (2017, 2021, 2022, and 2023) is largely consistent with the distinct meteorological conditions of each growing season (Porter and Gawith 1999). To mechanistically understand these differences, the growing season can be partitioned into three critical windows: the establishment and spikelet initiation phase (April–May), the stem elongation and flowering phase (June), and the grain-filling phase (July–August) (Acevedo et al. 2002). This period covers key developmental stages from tillering and spikelet initiation (double ridge to terminal spikelet, DR–TS) through maturity, with some plasticity in trait determination extending between windows.

**Fig. 11:**
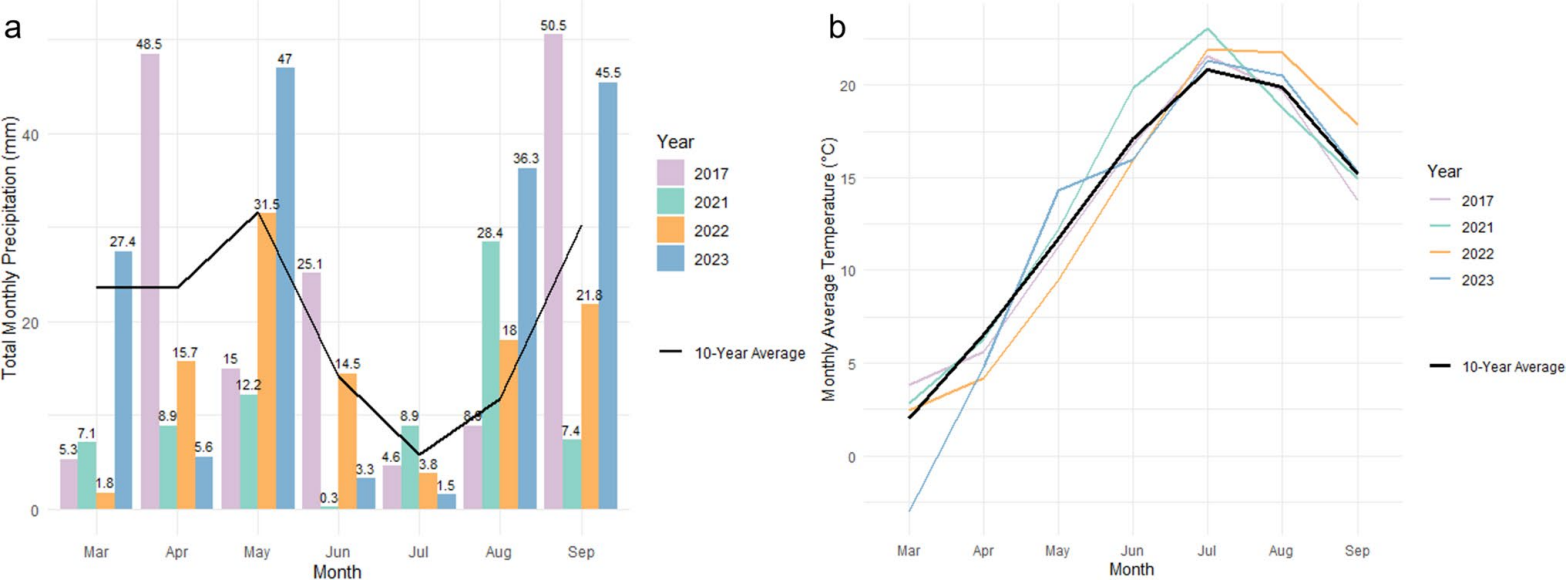
Seasonal climate during the spring-wheat growing season at Aberdeen, Idaho (Mar–Sep). **a** Aberdeen Monthly Precipitation (March–September): Total monthly precipitation (mm) for 2017, 2021, 2022, and 2023 (colored bars) with the 2015–2024 10-year monthly mean shown as a black line; values atop bars indicate month totals. **b** Aberdeen Monthly Average Temperature (March–September): Monthly mean air temperature (°C) for the same years (colored lines) with the 2015–2024 10-year monthly mean shown as a black line. These panels summarize the climatic context: cooler, wetter conditions in May 2023; pronounced early-summer drought in 2021; and warmer-than-average July–August in 2022—patterns used to interpret inter-annual variation in tSNS, HD, PHT, and TKW. Data source: NCEI, Aberdeen weather station (USC00100031)

The tSNS is primarily set during the DR–TS formation stage (April–May) (Rawson 1970). Conditions during this phase varied starkly across years, likely contributing to the observed range in tSNS. For instance, the cool and exceptionally moist conditions in 2023 (where May rainfall was substantially above the decadal mean) likely provided a prolonged, low-stress period for spikelet differentiation, which is consistent with the highest observed tSNS (Fig. 11, Supplemental Table 4). In contrast, the onset of high-temperature stress in 2022 was likely associated with an increased abortion risk of spikelet primordia, contributing to the lowest tSNS (Barnabás et al. 2008; Jacott and Boden 2020).

PHT and HD are outcomes of cumulative development through the April–June period and are highly sensitive to both temperature-driven growth rates and water availability (Kronenberg et al. 2021; Bloomfield et al. 2023; Duvnjak et al. 2023). The severe water deficit in 2021, especially in June, is consistent with an accelerated phenology (earlier HD) as a stress-escape response (Fig. 11, Supplemental Table 4), which also limited biomass accumulation and resulted in the shortest PHT (Shavrukov et al. 2017; Chowdhury et al. 2021).

TKW is a function of the rate and duration of the grain-filling period (July–August) (Sofield et al. 1977; Wardlaw and Moncur 1995; Baillot et al. 2018). The hot and dry conditions during this window in 2022, particularly the high July–August temperatures with limited late-season rainfall, are consistent with a high-temperature stress-induced reduction in the grain-filling duration, offering a potential explanation for the lower TKW (Dias and Lidon 2009). Conversely, the more moderate temperature profile during this period in 2023, coupled with a wetter-than-average August, likely facilitated a more complete grain-fill and the highest TKW, albeit with the trade-off of wetter late-season conditions (Fig. 11, Supplemental Table 4).

Given the strong genotype-by-environment (G × E) interaction detected in the BLUPs data (Supplemental  Table 4), future work should adopt multi-environment genomic selection models that explicitly include environmental covariates (e.g., precipitation and temperature) using reaction-norm or envirotyping-enabled frameworks (Jarquín et al. 2017; Crossa et al. 2022). A limitation of this study is the environmental scope: all data were collected at a single location across multiple years. Although this design captures interannual climatic variability, G × E arising from differences in latitude and sowing dates likely modulates the effects of *Ppd*, *VRN*, and *FT* alleles—effects that are expected to be larger in multi-location trials spanning diverse photothermal regimes. Accordingly, a broader multi-environment evaluation is a logical next step to quantify G × E and test these marker–environment links. Given the limited number of environments, we interpret these links as directional hypotheses rather than confirmed causal relationships. Ultimately, phenotype reflects the interplay of genotype (G), environment (E), and management (M).

### Comparative analysis of parametric and non-parametric models in GS for wheat yield improvement

The seven models compared in the present study, GBLUP, RR, BRR, LASSO, RKHS, RF, and SVM, differed in their assumptions and characteristics. The first four models are parametric, while the latter three are non-parametric (de Los Campos et al. 2013b; Howard et al. 2014; Montesinos López et al. 2022). A key finding of this study was the superior performance of the non-parametric models, RKHS and RF, over all parametric models tested for nearly all traits.

Generally, non-parametric models, such as RKHS and RF, outperform parametric models in prediction performance. This may be due to a couple of factors. First, non-parametric models can better capture complex genetic effects, such as epistasis, which are difficult to explicitly model using parametric methods (Howard et al. 2014). Second, the strict assumptions of parametric models (e.g., normality, linearity) are often violated in practice (Howard et al. 2014). Non-parametric models do not rely on these assumptions and can more flexibly capture the relationships within the data, resulting in models that better fit diverse populations. In the present study, the RF and RKHS models outperformed other models for almost all traits (Supplemental Table 8). RKHS has been extensively studied and partially applied in production and it was found to outperform traditional models in most cases (Jacquin et al. 2016). The International Maize and Wheat Improvement Center (CIMMYT) successfully implemented RKHS for predicting grain yield in wheat, the RKHS model effectively differentiated high- and low-potential lines, showing a 7% estimated genetic gain between the top and bottom 20% of individuals ranked by their GEBVs for yield (Dreisigacker et al. 2021). Therefore, it was chosen as the optimal model for subsequent experiments.

The SVM model performed poorly for all traits, which is consistent with previous findings (Charmet et al. 2020). However, some studies have shown that SVM has relatively high predictive ability (Ogutu et al. 2011; Howard et al. 2014; Zhao et al. 2020). This contradiction possibly results from different choices of SVM model hyperparameters. The main challenge in obtaining a good SVM model lies in correctly setting the meta-parameters (Jacquin et al. 2016), which requires substantial computational time to thoroughly explore the entire hyperparameter space. This typically demands not only proficiency in programming and algorithms but also excellent computational resources. If all parameters are adjusted by searching through a typically large grid of values, the computational cost quickly becomes prohibitive (Lourenço et al. 2024). This high cost might be why SVM models have not been widely used to date.

Although non-parametric models have many advantages compared to parametric models, they cannot completely replace parametric models. Parametric models (such as GBLUP, RR) remain the most widely used GS models currently. Non-parametric models typically lack interpretability and usually do not provide inferences about the relative weights of different genomic regions (Howard et al. 2014). Traditional models, such as BRR, RR, and GBLUP, have numerous software options (e.g., BGLR, rrBLUP) that allow adjustments to different parts of the model. In contrast, while parametric models like RR also have hyperparameters (e.g., the penalty parameter λ), many non-parametric models involve tuning a larger set of more abstract hyperparameters that often lack direct biological significance, thus limiting their inference on the contribution of specific alleles to traits. What’s more, under certain conditions, the predictive ability of parametric models is like that of non-parametric models. For example, in a study by Joshi et al. (2024) of protein-related traits, RR-BLUP outperformed all other models for test weight prediction, including RF and RKHS. In fact, many different prediction equations produce approximately the same likelihood and minimum prediction error rate (de Los Campos et al. 2013b).

In conclusion, selecting a prediction model for GS is not a one-size-fits-all problem. Different models should be selected based on specific research objectives and the genetic architecture of the trait, no single model performs best in all situations. (Heslot et al. 2012; de Los Campos et al. 2013b; Desta and Ortiz 2014; Azodi et al. 2019).

### Enhancing predictive ability in wheat yield through integration of major gene markers as fixed effects

Previous studies have demonstrated that incorporating markers linked to major genes or QTLs as fixed effects in GS models can enhance predictive ability across various plant and animal species (Bernardo 2014; Moore et al. 2017; Li et al. 2019, 2024; Sarinelli et al. 2019; Kim et al. 2022). Our results confirmed this finding, showing significant improvements in predictive ability for yield-related traits, including HD, PHT, and tSNS, with the addition of just a single relevant marker (Fig. 9). These improvements align with the biological understanding of these traits, given the well-documented roles of photoperiod-related genes like *Ppd* and *FT* in the regulation of heading date and spikelet development pathways (Beales et al. 2007; Isham et al. 2021; Brassac et al. 2021; Chen et al. 2022). *Rht-D1*, a key gene from the "Green Revolution," significantly reduces plant height and improves lodging resistance (Pearce et al. 2011), while *Rht25b* has been reported to affect grain weight (Mo et al. 2018). These observations underscore the importance of incorporating relevant markers into GS models, as highlighted by Bernardo (2014), who proposed modeling known major genes or QTLs as fixed effects while treating unknown minor QTLs as random effects. Furthermore, the combination of multiple markers demonstrated even greater improvements in predictive ability across all traits compared to single-marker models. This finding is consistent with previous research, such as the work by Sarinelli et al. (2019). Under the breeder’s equation (R = $\frac{{i \cdot r \cdot \sigma_{A} }}{L}$) (Bernardo 2010; Cobb et al. 2019), if selection intensity (i), additive variance ($\sigma_{A}$), and cycle time (L) are held constant across models, the expected response (R) scales linearly with predictive accuracy (r). Thus, by boosting predictive accuracy r, the multi-gene fixed-effect strategy effectively intensifies selection (higher i·r), producing around 1.07–1.23 greater response across traits per cycle compared with the baseline 90K RKHS model and thereby speeding genetic improvement. (Supplementary Table 12).

Most current studies employ a two-step approach where significant SNPs are first identified through GWAS and then incorporated into GS models as fixed effects (Moore et al. 2017; Li et al. 2019; Sarinelli et al. 2019; Kim et al. 2022). However, this approach is only valid when the SNP identification is performed exclusively within the training set for each cross-validation fold to prevent data leakage and overfitting; otherwise, the predictive ability can be overestimated. When properly implemented, this approach is cost-effective as it leverages existing data to enhance genomic prediction without additional expense (Li et al. 2019), it has inherent limitations. Certain gene loci may be missed, especially in genomic regions that lack adequate marker coverage. This issue is particularly pronounced for complex traits with low heritability, such as grain yield (YLD) (Sarinelli et al. 2019). In contrast, Kompetitive Allele Specific PCR (KASP) markers allow for the targeted addition of genes and markers of interest into GS models. These targets are often well-characterized loci with known biological effects, identified through decades of classical genetic studies or QTL mapping. This approach can therefore capture the contributions of important genes that a GWAS might miss because they may not reach the stringent significance threshold in a particular population or study.

Our findings demonstrate that including known genes markers enhance the model’s predictive ability for complex, low-heritability traits like YLD. For instance, the markers Ppd-D1_1 and FTA2 individually increased predictive ability for YLD respectively by only 0.5% and 1.4%. However, when these markers were combined, the predictive ability improved by 10.1% (Supplemental Table 13). Research on these genes demonstrates their close interconnections. The photoperiod-insensitive allele of *Ppd-1* significantly influences the expression of *FT-1* genes. Studies have shown that wheat plants carrying the photoperiod-insensitive allele exhibit increased *FT-1* expression at night during short-day conditions compared to those with the photoperiod-sensitive (PS) allele (Boden et al. 2015). This observation may be due to misexpression of the *Ppd-1* gene at night, leading to elevated *FT* gene expression levels (Gauley and Boden 2021). All photoperiod-insensitive (PI) lines display similar misexpression patterns (Beales et al. 2007; Wilhelm et al. 2009; Díaz et al. 2012), indicating the close connection between *Ppd-1* and *FT-1* genes. Furthermore, wheat possesses three copies of *FT-1*: *FTA1*, *FTB1*, and *FTD1* (Chen et al. 2020, 2022; Brassac et al. 2021), with *FTD1* potentially associated with yield-related quantitative trait loci (QTLs) (Isham et al. 2021). *FT-2*, the closest paralog to *FT-1* in wheat with 78% protein similarity (Lv et al. 2014; Shaw et al. 2019; VanGessel et al. 2022), suggests a specific role in wheat development.

Our analysis of the variance explained (R^2^) by individual markers and their combination supports the presence of interactions among the underlying adaptation-related genes. We assessed the joint main effects by One-way ANOVA model, comparing a model with the four markers coded as fixed additive effects to an intercept-only model (Supplementary Table 13). While individual markers such as PpdD1_1, FTB1, FTA2, and FTD1 explained only a small proportion of the variance in YLD (R^2^ values of 0.0005, 0.0139, 0.014, and 0.0585, respectively), the combined effects explained a higher proportion of variance (R^2^ = 0.101, Supplementary Table 13).

It is important to note, however, that the selection of these trait-specific marker combinations was performed in a data-driven manner on the dataset used for model evaluation. While this approach clearly demonstrates the potential of leveraging major genes to improve predictive ability, the specific combinations identified here may be subject to a degree of overfitting. Therefore, the magnitude of the accuracy improvement should be interpreted with caution, and these specific marker sets warrant further validation in independent populations to confirm their robustness.

## Conclusion

This study assessed genomic prediction models for key agronomic traits in spring wheat across multiple environments. The RKHS model was the most effective, offering superior predictive abilities for complex traits influenced by numerous small-effect loci, especially when using BLUPs to integrate phenotypic data. Incorporating major genes as fixed effects into the RKHS model enhanced predictive ability, with trait-specific combinations outperforming single-marker models. Notably, integrating multiple relevant major genes outperformed single-gene approaches, emphasizing the importance of key genetic factors in GS. Adding population structure as a fixed effect had little influence on accuracy. Overall, these findings demonstrate that combining major genes as fixed effects in GS models significantly improves the prediction of complex traits, offering valuable insights for wheat breeding programs aiming to enhance yield and other important characteristics. Future research should focus on integrating additional adaptive traits, exploring novel markers, and optimizing GS models to address emerging challenges in wheat production, such as climate resilience. By adopting these strategies, breeding programs can achieve faster genetic gains and develop high-performing, resilient wheat varieties to meet the demands of a growing population.

## Supplementary Information

Below is the link to the electronic supplementary material.

Supplementary file1 (XLSX 4530 kb)
